# Reprogramming barriers and enhancers: strategies to enhance the efficiency and kinetics of induced pluripotency

**DOI:** 10.1186/s13619-015-0024-9

**Published:** 2015-11-11

**Authors:** Behnam Ebrahimi

**Affiliations:** Yazd Cardiovascular Research Center, Shahid Sadoughi University of Medical Sciences, Yazd, Iran

**Keywords:** Cellular reprogramming, iPSCs, Mbd3, C/EBPα, p53

## Abstract

Induced pluripotent stem cells are powerful tools for disease modeling, drug screening, and cell transplantation therapies. These cells can be generated directly from somatic cells by ectopic expression of defined factors through a reprogramming process. However, pluripotent reprogramming is an inefficient process because of various defined and unidentified barriers. Recent studies dissecting the molecular mechanisms of reprogramming have methodically improved the quality, ease, and efficiency of reprogramming. Different strategies have been applied for enhancing reprogramming efficiency, including depletion/inhibition of barriers (p53, p21, p57, p16^Ink4a^/p19^Arf^, Mbd3, etc.), overexpression of enhancing genes (e.g., *FOXH1*, *C/EBP alpha*, *UTF1*, and *GLIS1*), and administration of certain cytokines and small molecules. The current review provides an in-depth overview of the cutting-edge findings regarding distinct barriers of reprogramming to pluripotency and strategies to enhance reprogramming efficiency. By incorporating the mechanistic insights from these recent findings, a combined method of inhibition of roadblocks and application of enhancing factors may yield the most reliable and effective approach in pluripotent reprogramming.

## Introduction

Somatic cells can be reprogrammed into induced pluripotent stem cells (iPSCs) by ectopic expression of a set of core pluripotency-related transcription factors [1–3], most commonly consisting of Oct4, Sox2, Klf4, and c-Myc (OSKM). iPSCs can provide a valuable patient-specific cell source for regenerative medicine, drug discovery, and disease modeling. These cells and their production have attracted enormous scientific and public interest. However, reprogramming is a time-consuming process and suffers from low efficiency. These features are regarded as limitations for clinical applications of iPSCs [1–6]. Therefore, a greater understanding of the reprogramming process will assist identification of reprogramming roadblocks and more efficient reprogramming technologies. There are emerging tactics to increase the efficiency of reprogramming by removing barriers. A growing list of small molecules, miRNAs, siRNAs, and growth factors has been reported to boost reprogramming efficiency or substitute reprogramming transcription factors. Typically, these enhancing strategies include inhibition of genetic or epigenetic barriers of reprogramming [7–11], overexpression of certain transcription factors, and administration of special small molecules and cytokines [12–18]. Moreover, the species and tissue origins of donor cells have significant effects on reprogramming efficiency and kinetics [19]. Indeed, distinct cell types appear to have different requirements for enhancement in their reprogramming efficiency [20]. Thus, different strategies may need to be adopted for efficient reprogramming of different cell types. Mouse and human fibroblasts are the most commonly used primary cell sources in cellular reprogramming studies. The current review describes different factors that can act as barriers or enhancers during pluripotent reprogramming regardless of a specific cell source or species.

Highly efficient and rapid direct reprogramming methods for the generation of iPSCs and other desired cell types may considerably improve the prospects for certain approaches, such as cell transplantation therapies and direct in vivo reprogramming. Here, the methods that can remove or overcome reprogramming barriers and enhance reprogramming efficiency are discussed (Table 1).

**Table 1:** Different barriers and enhancers of reprogramming

Enhancing strategies		Enhancers
	Activation of enhancers	C/EBPα [12]; UTF1 [53, 60]; Mef2c [60]; Tdgf1 [60]; FOXH1 [25]; GLIS1 [24]; mutated reprogramming factors [33, 35, 36]; MDM2 [56]; Bcl-2 [52]; CCL2 [14]; Kdm3a, Kdm3b, Kdm4c, and Kdm4b/2b [81]; Jhdm1a/1b [81, 87]; MOF [97]; Mbd3 [13, 104] (or their small molecule activators); Wnt/β-catenin signaling [20]; small molecule Pitstops 1 and 2 [80]; vitamin C [81].
		Barriers
	Inhibition of barriers	p53 [7, 11, 29, 40, 44, 52–63], p57 [7], p38 [50], p16^Ink4a^/p19^Arf^, [52, 57, 58] p21^Cip1^ [7, 11, 29, 40, 44, 52–63].
		TGF-β [20, 40, 80], MAP kinase [20], Aurora A kinase [50], MEK/ERK [20, 46, 47], Gsk3, Wnt/β-catenin signaling pathways [69, 70], LATS2 [77], PKC [49], IP3K [50].
		Native/somatic gene or transcriptional regulatory network (GRN/TRN) [115, 124, 125].
		Specific members of ADAM family (e.g., ADAM7, ADAM21, ADAM29), endocytosis: (e.g., DRAM1, SLC17A5, ARSD), phosphatase: (e.g., PTPRJ, PTPRK, PTPN11) [80].
		Chromatin regulators: (e.g., ATF7IP [80], MacroH2A [85, 86], Mbd3 (?) [8, 9, 103, 105, 106], Setdb1a [81].
		Transcription factors: (e.g., TTF1, TTF2, TMF1, T [80]), Bright [23].
		Fbxw7 (a member of ubiquitin-proteasome system (UPS)) [78]
		Lzts1, Ssbp3, Arx, Tfdp1, Nfe2, Ankrd22, Msx3, Dbx1, Lasp1, and Hspa8 [60].

Notably, inhibition/depletion of barriers is considered as enhancing strategy

## Transcription factors affecting reprogramming efficiency

Forced expression of several known transcription factors drives pluripotent reprogramming of somatic cells [1–3, 5, 21, 22]; however, there are some transcription factors that can act as enhancers or barriers during reprogramming [23–25] (Table 1).

### The Gli-like transcription factor GLIS1

GLIS1 is a Gli-like transcription factor that has been recognized as an enhancer of reprogramming, and it can effectively and specifically promote iPSC generation from both mouse and human fibroblasts when co-expressed with OSK, in a p53-independent mechanism and by activating several pro-reprogramming pathways. Moreover, GLIS1 physically interacts with OSK to assist the activation of reprogramming target genes. In addition, GLIS1 together with OSK can increase embryonic stem-cell-like (ESC-like) colony formation from human fibroblasts approximately up to 2-fold and 30-fold relative to OSKM and OSK, respectively [24].

### The forkhead box protein H1 (FOXH1)

Yamanaka’s group recently demonstrated that transcription factor forkhead box H1 (FOXH1) can facilitate iPSC generation (~15-fold) when together with OSKM is transduced into human adult fibroblasts. Interestingly, FOXH1 facilitates reprogramming process by promoting mesenchymal-to-epithelial transition (MET) of TRA-1-60^+^ intermediate reprogrammed cells. Moreover, inhibition of FOXH1 during reprogramming can block iPSC generation [25]. It has been revealed that unlike GLIS1 that facilitates earlier stages, FOXH1 improves reprogramming efficiency by acting at the late stages [24, 25]. Together, these data highlight the important role of FOXH1 during the reprogramming process.

### The Bright/Arid3A transcription factor

Bright/Arid3A is a member of the ARID family of DNA-binding transcription factors [26, 27]. Depletion of *Bright*/*Arid3A* can confer an increased developmental plasticity and expression of core pluripotency genes to both human and mouse somatic cells, representing this differentiation hallmark as a suppressor of lineage plasticity [28]. Furthermore, Bright/Arid3A has been recently identified as a mouse reprogramming barrier by its direct binding to the promoter/enhancer regions of *Oct4*, *Sox2*, and *Nanog* repressing these genes. Popowski et al. revealed that the depletion of *Bright* improves reprogramming efficiency of mouse embryonic fibroblasts (MEFs) 15- to 40-fold [23]. Moreover, they showed that *Bright* depletion allows reprogramming in the absence of Sox2 and Klf4 but not Oct4. Surprisingly, *Bright*-deficient MEFs can spontaneously form stable iPSCs in leukemia inhibitory factor (LIF)-containing medium and in the absence of reprogramming factor expression [23]. In summary, depletion of Bright improves reprogramming efficiency through bypassing senescence, promoting self-renewal, antagonizing differentiation, and direct derepression of pluripotency factors [23].

### Engineered factors

Oct4 and Sox2 are essential transcription factors for pluripotent reprogramming, and their interaction is fundamentally important [29–34]. Thus, enhancing the potency of these factors to induce pluripotency will be of interest. Remarkably, Wang et al. demonstrated that the fusion of the VP16 transactivation domain to OCT4, NANOG, and SOX2 converts them into more efficient factors. These factors can reprogram mouse and human fibroblasts into iPSCs with enhanced efficiency and accelerated kinetics [35]. Interestingly, the Stanton and Kolatkar groups indicated that a single amino acid replacement in the HMG domain of Sox7 and Sox17 that mediates Oct4 interaction transforms these endoderm-promoting factors into pluripotent reprogramming factors [33, 36]. They showed that engineered Sox variants (Sox7EK and Sox17EK) cannot only replace Sox2 but also induce pluripotency in MEFs even five to seven times more efficient than normal Sox2 [33]. These mutated Sox factors can reprogram human cells into iPSCs with accelerated kinetics and more efficiently than Sox2 [33]. Moreover, overexpression of these factors can confer LIF resistance to mouse embryonic stem cells (mESCs) [33]. Therefore, reengineered factors with enhanced transcriptional potency can promote reprogramming efficiency and broaden our understanding of transcription-factor-mediated reprogramming of somatic cells.

## Epithelial-to-mesenchymal transition (EMT) and mesenchymal-to-epithelial transition (MET)

Pluripotent reprogramming of somatic cells represents and requires mesenchymal-to-epithelial transition (MET) that is coordinated by suppression of pro-epithelial-to-mesenchymal transition (EMT) signals [37–39]. MET is promoted by BMP-Smad signaling-mediated upregulation of miR-205 and miR-200 and is required for and enhances mouse pluripotent reprogramming in the initiation phase [40]. In contrast, EMT, the opposite of MET, is a developmental process that represents a differentiation process in stem cell and developmental biology; for instance, fibroblasts are products of EMT. Transforming growth factor-β (TGF-β) signaling has a critical and dominant role in EMT [37, 38]. It has been shown that TGF-β signaling is a barrier of mouse and human reprogramming and its inhibition can enhance reprogramming [38, 41–43] and also can replace c-Myc or Sox2 in mouse [38, 41, 42]. In addition to the discussions above, p53, a known reprogramming roadblock, inhibits the reprogramming process in the early stages by inhibiting MET [44].

Interestingly, iPSC transcription factors progress the reprogramming process by initiating the MET program (e.g., inducing E-cadherin) and shutting down EMT by diminishing intrinsic barriers (e.g., Snail, TGF-β1/TGF-βR2) [38]. Consequently, EMT and its main regulator, TGF-β signaling, are barriers of mouse and human pluripotent reprogramming, and their inhibition can enhance reprogramming [38, 41–43]. By contrast, Unternaehrer et al. reported that EMT factor SNAI1 (SNAIL) overexpression can paradoxically enhance reprogramming efficiency in human cells and in mouse cells, depending on the strain [45]. Therefore, reprogramming efficiency can be improved by preventing EMT and activating MET, but regarding unexpected data, more investigation is needed to elucidate the exact roles of MET and EMT during the trajectory of reprogramming.

## Barrier kinases

Several kinase enzymes have been recognized as reprogramming barriers. It has been shown that the chemical inhibition of two kinases of mitogen-activated protein kinase/extracellular signal regulated kinases 1 and 2 (Mek/Erk) and glycogen synthase kinase 3 (Gsk3) in cooperation with LIF (2i/LIF) can provide an optimum culture condition for the maintenance of ground-state pluripotency in mESCs [46]. Moreover, 2i/LIF promotes the maturation step (conversion of pre-iPSC into iPSC) and transition to ground-state pluripotency during mouse reprogramming by activating *Nanog* [47, 48]. Likewise, 2i/LIF in combination with a small molecule inhibitor of the protein kinase C (PKC) (Gö6983) can favor the induction of ground-state pluripotency in human pluripotent stem cells [49]. Mitogen-activated protein (MAP) kinase is also a reprogramming barrier, and its inhibition can facilitate pluripotent reprogramming of MEFs [20, 47]. In addition, further kinases, including p38, inositol trisphosphate 3-kinase (IP3K), and Aurora A kinase, have been identified as reprogramming barriers. Li et al. indicated that small molecule-mediated inhibition of these kinases can potently enhance iPSC generation from MEFs [50]. Altogether, these findings represent inhibitory roles of specific kinases (e.g., Aurora A, PKC, MEK, and Gsk3) during reprogramming and especially in maturation of iPSCs.

## Barrier signaling pathways

### The p53-p21 pathway

Different signals can activate p53 that has key roles in the regulation of apoptosis, induction of cell cycle arrest, senescence, and differentiation [51]. It has been demonstrated that overexpressed factors (i.e., OSKM), individually or in combination, can strongly activate the p53 pathway during reprogramming. This pathway impedes the reprogramming process and causes a dramatic reduction in reprogramming efficiency [52].

Several groups have shown that the p53-p21 pathway is an inhibitor of both human and mouse somatic cell reprogramming and that pluripotent reprogramming can be done more efficiently and with accelerated kinetics in the absence of p53 [7, 11, 29, 40, 44, 52–63]. These studies have indicated that direct suppression of the p53 signaling pathway increases the reprogramming efficiency of distinct cell types between 10-fold and 100-fold.

It has been revealed that reprogramming efficiency is sensitive to p53 protein dosage, and even low levels of its activity compromise the reprogramming process [52]. Utikal and colleagues showed that secondary OSKM doxycycline-inducible MEFs derived from p53 knockout iPSCs acquire a reprogramming efficiency of about 80 % [58] that is striking and indicative of a potent inhibitory role of the p53 pathway during pluripotent reprogramming. However, it is noteworthy that the results from secondary systems or genetically manipulated cells cannot be used in clinical applications in humans.

Remarkably, p53 restricts reprogramming of mouse and human cells not only by decreasing reprogramming efficiency and kinetics [52, 53, 56–59] but also by eliminating DNA-damaged cells at the early stages of the reprogramming process via apoptosis [55]. Moreover, p53 depletion allows efficient reprogramming in the absence of c-Myc [56] and c-Myc/Klf4 [52]. Similarly, the *Ink4/Arf* locus which encodes three transcripts of p16^Ink4a^, p19^Arf^, and p15^Ink4b^ tumor suppressors acts as a main barrier of pluripotent reprogramming in both human and mouse somatic cells by activation of the p53-p21 pathway. Inhibition of the *Ink4/Arf* products increases the efficiency of reprogramming [52, 57, 58]. Indeed, inactivation or suppression of the *Ink4a/Arf*-p53 pathway (a key senescence pathway) removes a key roadblock of pluripotent reprogramming and acquisition of immortality [58].

Regarding the roles of the p53 pathway in pluripotent reprogramming, it has been shown that the ectopic expression of MDM2, a negative regulator of p53, can mimic *p53* suppression [56]. In addition, overexpression of the proto-oncogene Bcl-2, an anti-apoptotic protein, increases the frequency of mouse iPSC formation by fourfold [52]. On the other hand, knockout or prolonged suppression of p53 reduces the quality of iPSCs and can lead to genetic instability [56, 64–66]. Moreover, knockout of p53 can cause or increase chromosome end-to-end fusions and chromosomal breaks in the reprogrammed MEFs compared to wild-type iPSCs [55]. Accordingly, p53 as a main roadblock of pluripotent reprogramming decreases the quantity of reprogrammed cells; however, it can increase the quality of produced iPSCs by the induction of apoptosis in suboptimal cells, elimination of these cells, and subsequently preventing them from becoming iPSCs. Interestingly, transient suppression of p53 can significantly improve the efficiency of human somatic cell reprogramming [29, 62, 63]. Rasmussen et al. recently indicated that non-integrative reprogramming approaches in combination with transient p53 inhibition allow efficient reprogramming without excessive DNA damage due to the presence of low levels of p53 and a reasonable activity of the apoptotic pathway [29]. Remarkably, the main consequence of this strategy is genomic stability of generated iPSCs without any significant effect on apoptosis and DNA damage [29]. Collectively, inhibition of the p53 pathway by small molecules, transiently and in a reversible manner, could be a useful tool for enhancing reprogramming efficiency. For instance, the small molecule Pifithrin-α as a p53 inhibitor [67] can be used for transient inhibition of the p53 pathway and enhancing reprogramming efficiency without further genetic instability and malignancies that may arise due to prolonged inhibition of p53 in resultant iPSCs.

### Wnt/β-catenin, TGF-β, and Hippo signaling pathways

The Wnt/β-catenin signaling pathway has differential roles during different stages of direct and cell-fusion-mediated mouse reprogramming, and temporal modulation of this pathway can considerably increase the efficiency of reprogramming [68–71]. Indeed, repression of Wnt/β-catenin signaling in the early stages of reprogramming followed by its normal activity in the later stages can significantly enhance the process [69, 70]. This role of the Wnt/β-catenin pathway during mouse reprogramming is almost opposite of its role during cardiomyocyte derivation from the human pluripotent stem cells that needs activation of Wnt/β-catenin in the early stages and inhibition of this pathway in the late stages of differentiation [72, 73]. Interestingly, findings suggest that the temporally differential behavior of the Wnt/β-catenin pathway is consistent with the activation of MET during the establishment of pluripotency and also the activation of EMT during differentiation [37, 70]. Furthermore, Murayama et al. recently reported that the inhibition of Wnt signaling can significantly increase the efficiency of mouse epiblast stem cell (EpiSC) conversion to naïve-like pluripotent stem cells in response to LIF, an effect similar to the overexpression of E-cadherin [74].

Conversely, it has been recently indicated that the administration of ascorbic acid together with the inhibition of TGF-β and activation of Wnt/β-catenin signaling pathways can induce an approximately non-stochastic and highly efficient (80–95 %) OSKM-mediated reprogramming in both somatic and progenitor cells (i.e., MEFs, hepatoblasts, and blood progenitors) in a rapid, synchronous, and homogeneous manner [20]. In addition, Stadtfeld and colleagues showed that the activation of Wnt/β-catenin signaling and the inhibition of the TGF-β pathway are sufficient for enhancing the reprogramming efficiency of granulocyte monocyte progenitors (GMPs) and hepatoblasts, respectively [20]. Thus, more research is needed to elucidate some discrepancies from different reports regarding the roles of signaling pathways in pluripotent reprogramming.

Another barrier pathway is Hippo signaling that has critical roles in tumor suppression and stem cell function. Interestingly, modulation of this pathway has beneficial effects in anticancer therapeutic strategies and also stimulating tissue repair and regeneration [75]. This pathway has distinct roles in human and mouse iPSC generation [76, 77]. In detail, LATS2 a serine/threonine protein kinase of the Hippo pathway can act as a roadblock in human reprogramming, and its inhibition can improve the efficiency of reprogramming (~2.5-fold) [77].

## The ubiquitin-proteasome system (UPS)

Buckley et al. indicated that silencing of E3 Ligase Fbxw7, a member of the ubiquitin-proteasome system (UPS), enhances pluripotent reprogramming of MEFs (twofold) and impedes differentiation of mESCs through ubiquitination and stabilization of c-Myc [78]. Moreover, *Fbxw7* siRNA can replace exogenous c-Myc expression whereas it can concomitantly enhance the efficiency [78]. Collectively, recent findings have demonstrated that the ubiquitin-proteasome system as a common posttranslational modification has important roles in the maintenance of pluripotency in mouse and human ESCs [78, 79] as well as pluripotent reprogramming [78].

## Insights gained from functional genomics and genome-wide studies

In addition to the different factors that are discussed above, Song and colleagues recently reported the identification of distinct sets of barriers to human pluripotent reprogramming using a novel approach allowing genome-wide screens at an unprecedented scale [80]. They found 956 genes as barriers to reprogramming using their integrative approach [80]. However, among these large numbers of genes, several are more effective in hampering reprogramming. The products of these genes are involved in different cellular processes, including transcription (TTF1, TTF2, TMF1, T), chromatin regulation (ATF7IP, ARID4A, CENPB, MED19), ubiquitination (UBE2D3, UBE2E3, RNF40), dephosphorylation (PTPRJ, PTPRK, PTPN11), endocytosis and vesicular transport (DRAM1, SLC17A5, ARSD), and cell adhesion/motility (ADAM7, ADAM21, ADAM29) [80]. Their results of multiple- and single-gene(s) inhibition have shown significant increases (1.5-fold–15-fold) in reprogramming efficiency [80].

In line with this approach, Yang et al. recently defined four critical steps in mouse pluripotent reprogramming from initiation to maturation by appropriate markers and applying fluorescence-activated cell sorting (FACS) [60]. Remarkably, using a genome-wide RNA interference (RNAi) screen and integrated transcriptome analysis, they identified key regulatory genes at each transition step of reprogramming. Their findings suggest the transition from Thy1^−^ into SSEA1^+^ cell state as a rate-limiting step, whereas expression of pluripotency factors are needed for overcoming this stage [60].

Interestingly, Yang et al. demonstrated that non-differentially expressed genes can act as enhancers (e.g., *Mef2c*, *Utf1*, or *Tdgf1*) or barriers (e.g., *Lzts1*, *Ssbp3*, *Arx*, *Tfdp1*, *Nfe2*, *Ankrd22*, *Msx3*, *Dbx1*, *Lasp1*, and *Hspa8*) during different steps of reprogramming. For instance, of these non-differentially expressed genes, inhibition of *Nfe2* or *Msx3* can enhance iPSC generation about fivefold whereas in the same condition p53 knockdown can enhance threefold [60]. Furthermore, inhibition of *Hspa8* and *Lasp1* which act as barriers to reprogramming in the maturation step can enhance iPSC formation by 8- and 12-fold, respectively [60].

## Epigenetic factors and epigenetic modifications affecting reprogramming

### Histone H3 lysine 9 (H3K9) methylation

Histone H3 lysine 9 (H3K9) methylation at core pluripotency genes is an epigenetic barrier of mouse pluripotent reprogramming. It has been demonstrated that this barrier acts during the maturation and stabilization steps and traps reprogramming products in the pre-iPSC stage [81]. Moreover, it has been revealed that the Setdb1 histone methyltransferase blocks mouse reprogramming at the pre-iPSC stage in a BMP-dependent manner. Inhibition of *Setdb1* can promote conversion of pre-iPSCs into iPSCs with 100 % efficiency in the presence of vitamin C [81]. However, this is inconsistent with the result of *SETDB1* inhibition during early human reprogramming [82]. Interestingly, vitamin C can decrease H3K9 methylation at the pluripotency loci through the activation of histone demethylases (e.g., *Kdm3a*, *Kdm3b*, *Kdm4c*, and *Kdm4b*) to further improve reprogramming [81]. Furthermore, overexpression of histone demethylase *Kdm4b* can efficiently promote maturation and generation of iPSCs by removing H3K9me3 and H3K9me2. It is indicative of its rate-limiting activity during complete conversion of pre-iPSCs into iPSCs [81].

### Histone H3 lysine 79 (H3K79) methylation

H3K79 dimethylation (H3K79Me2) and H3K27 tri-methylation (H3K27me3) denote transcriptionally active and silenced genes, respectively. Moreover, lineage-specific transcriptional programs act as barriers to reprogramming to pluripotency [83, 84]. Onder et al. demonstrated that the active H3K79me2 mark acts as a roadblock during reprogramming by hindering repression of lineage-specific programs [82]. They displayed that the suppression of histone methyltransferases SUV39H1, YY1, and DOT1L enhances reprogramming efficiency [82]. Furthermore, it has been revealed that the small molecule or siRNA-mediated inhibition of DOT1L, a histone H3 lysine 79 methyltransferase, can induce a threefold to sixfold increase in efficiency of mouse and human somatic cell reprogramming [82]. Mechanistically, inhibition of DOT1L enhances reprogramming by removing the active H3K79me2 mark, increasing the repressive H3K27me3 mark at fibroblast genes, silencing the somatic program, and by a concomitant reverse action on the pluripotency-related genes [82]. In addition, it has been indicated that the histone variant macroH2A, a differentiation mark that at least in part contributes to the deposition of H3K27me3, is a barrier of reprogramming and that inhibition of macroH2A.1 and macroH2A.2 isoforms can significantly increase the efficiency of reprogramming [85, 86].

### H3K36me2/3 marks

It has been indicated that methylation at histone H3 lysine 36 (H3K36me2/3) acts as a roadblock during reprogramming and that elimination of the H3K36me2/3 histone marks in some loci is necessary for progression of reprogramming [20, 87]. Wang et al. revealed that vitamin C improves reprogramming efficiency and kinetics by enhancing the removal of these marks via Jumonji histone demethylases Jhdm1a/1b [87]. Moreover, overexpression of *Jhdm1b* (also known as *Kdm2b*) in conjunction with the inhibition of MAP kinase signaling enhances efficiency and synchronicity of mouse reprogramming [20].

Mechanistically, Jhdm1b enhances proliferation of fibroblasts and overcomes cellular senescence by removing the H3K36me2/3 marks at the *Ink4/Arf* locus and the subsequent suppression of this locus that is a known reprogramming barrier [87–89]. Moreover, Jhdm1b removes H3K36me2/3 histone marks from the promoter of microRNA (miRNA) cluster 302/367 to activate these miRNAs as facilitators of reprogramming and subsequently improves the efficiency of reprogramming [87, 90, 91]. These findings delineate, at least in part, the underlying mechanisms by which Jhdm1b/vitamin C can enhance the reprogramming process and represent the contribution of the H3K36 histone modification in both gene activation and suppression during efficient pluripotent reprogramming [87].

### Histone deacetylation

On the role of epigenetic modifications in reprogramming, it has been shown that histone deacetylation impedes reprogramming and that inhibition of histone deacetylase enzymes can enhance iPSC generation [30, 92, 93]. For instance, histone deacetylase (HDAC) inhibitors, including valproic acid [30, 94], butyrate [92, 93, 95], trichostatin A, and suberoylanilide [94], can improve reprogramming efficiency by facilitating epigenetic remodeling during pluripotency acquisition. It has been revealed that histone acetyltransferase (HAT) MOF (males absent on the first) is a key component of the ESC core transcriptional network. This enzyme regulates Wdr5 recruitment and H3K4 methylation at key regulatory loci in ESCs [96]. Moreover, MOF activity is required in the initial stages of reprogramming. Mu et al. indicated that the ectopic expression of MOF together with OSKM improves the reprogramming efficiency of human fibroblasts, while knockdown of MOF suppresses iPSCs production [97]. Indeed, MOF recruits Wdr5 to interact with the Oct4 promoter and to reactivate the expression of endogenous Oct4 [97].

### The Mbd3/NuRD complex

Reprogramming is principally an epigenetic process, and chromatin modifiers have critical roles in genome remodeling. In addition to the current study, epigenetic changes that facilitate iPSC reprogramming have been reviewed by Buganim et al. [98] and others [82, 99, 100]. Efficiencies of direct reprogramming into specialized cells (e.g., induced neurons >20 % [101] and induced cardiomyocytes = 20 % [102]) have been reported higher than iPSC production (<0.1 % [1]). These data suggest that the epigenetic state of starting cells can act as a reprogramming barrier. Fidalgoa et al. showed that the pluripotency-related transcription factor Zfp281 directly recruits the NuRD repressor complex to the *Nanog* locus and subsequently restricts *Nanog* reactivation and inhibits iPSC formation [103]. This finding shows that the NuRD complex has an inhibitory role in pluripotent reprogramming. Accordingly, Luo et al. showed that the methyl-CpG-binding domain protein 3 (Mbd3), a subunit of the NuRD, impairs pluripotent reprogramming of MEFs and that its inhibition improves reprogramming efficiency even in the absence of c-Myc or Sox2 [8]. They indicated that Mbd3 downregulates pluripotency-specific genes (*Nanog*, *Oct4*, and *Sox2*) and its depletion results in upregulation of these genes and improvement of reprogramming efficiency [8].

Confirming these findings, Rais et al. recently indicated that Mbd3 is a key molecular barrier preventing the deterministic induction of ground-state pluripotency [9]. They revealed that inhibition of *Mbd3* increases the efficiency of EpiSCs reversion into naïve pluripotent cells up to 80 % [9]. Moreover, *Mbd3*-depleted MEFs in an optimized reprogramming condition (2i/LIF) were reprogrammed into iPSCs with 100 % efficiency by day 8 in comparison with the 20 % reprogramming efficiency in wild-type MEFs in the same condition [9]. Remarkably, adult progenitor cells, including common myeloid progenitor cells (CMPs), hematopoietic stem cells (HSCs), neural precursor cells (NPCs), and also terminally differentiated cells (i.e., monocytes and mature B and T cells), were reprogrammed with 100 % efficiency in this condition [9]. However, the roles of GSK3β and MEK signal inhibition and LIF are undeniable in this reprogramming cocktail. Surprisingly, in an apparent disagreement with the findings of Fidalgoa et al. [103], Luo et al. [8], and Rais et al. [9], dos Santos et al. recently reported the generation of reprogramming intermediates or pre-iPSCs from *Mbd3* ^−/−^ neural stem cells (NSCs) by c-Myc, Klf4, and Oct4 (MKO) but less efficient and with delayed kinetics [13]. However, the inhibitory role of serum in the dos Santos et al. reprogramming medium is undeniable as demonstrated earlier [81]. Furthermore, Silva’s group revealed that Mbd3 depletion strongly impairs conversion of NSCs into pre-iPSCs in the initiation phase of reprogramming and also strongly reduces the efficiency of conversion to naïve pluripotency [13]. They demonstrated the requirement of Mbd3 in the initiation phase, but not in the establishment of pluripotency during NSC reprogramming [13]. This is to the contrary of Rais et al. [9] that reported the inhibitory activity of Mbd3 before the establishment of pluripotency in the early stages. Moreover, *Mbd3* ^−/−^ iPSCs showed slower proliferation and impaired embryoid body differentiation in the dos Santos et al. reprogramming system [13]. Additionally, they indicated that *Mbd3* depletion impairs reversion of EpiSCs to naïve pluripotency [13]. Collectively, dos Santos et al. concluded that depletion of Mbd3/NuRD cannot enhance reprogramming efficiency of MEFs, and moreover, its overexpression has no positive or negative effect on the efficiency, depending on the reprogramming context [13]. These findings are in apparent disagreement with the results of Rais et al. [9], Luo et al. [8], and Fidalgoa et al. [103]. Furthermore, some recent studies have not found an inhibitory role for Mbd3 during mouse and human reprogramming using genome-wide RNAi screen [60, 80] and selective RNAi screen [82]. Regarding these discrepancies, Silva and colleagues [104] raised a concern that there are some methodological issues in the study of Rais et al. [9] that negatively affect accurate interpretation of the results. Thence, Hanna and colleagues [105] in disagreement with Bertone et al. [104] confirmed the inhibitory role of the Mbd3/NuRD pathway in the maintenance and induction of pluripotency by providing new data [105]. Most recently, Wernig and colleagues independently confirmed the validity and authenticity of the Rais et al. method and deterministic reprogramming [106]. This topic has recently attracted much attention and has become a controversial issue in the field of reprogramming. Thus, further investigations are needed to elucidate the basis of the contradicting and striking findings of these groups.

#### Molecular mechanisms underlying Mbd3 interactions

**Fig. 1:**
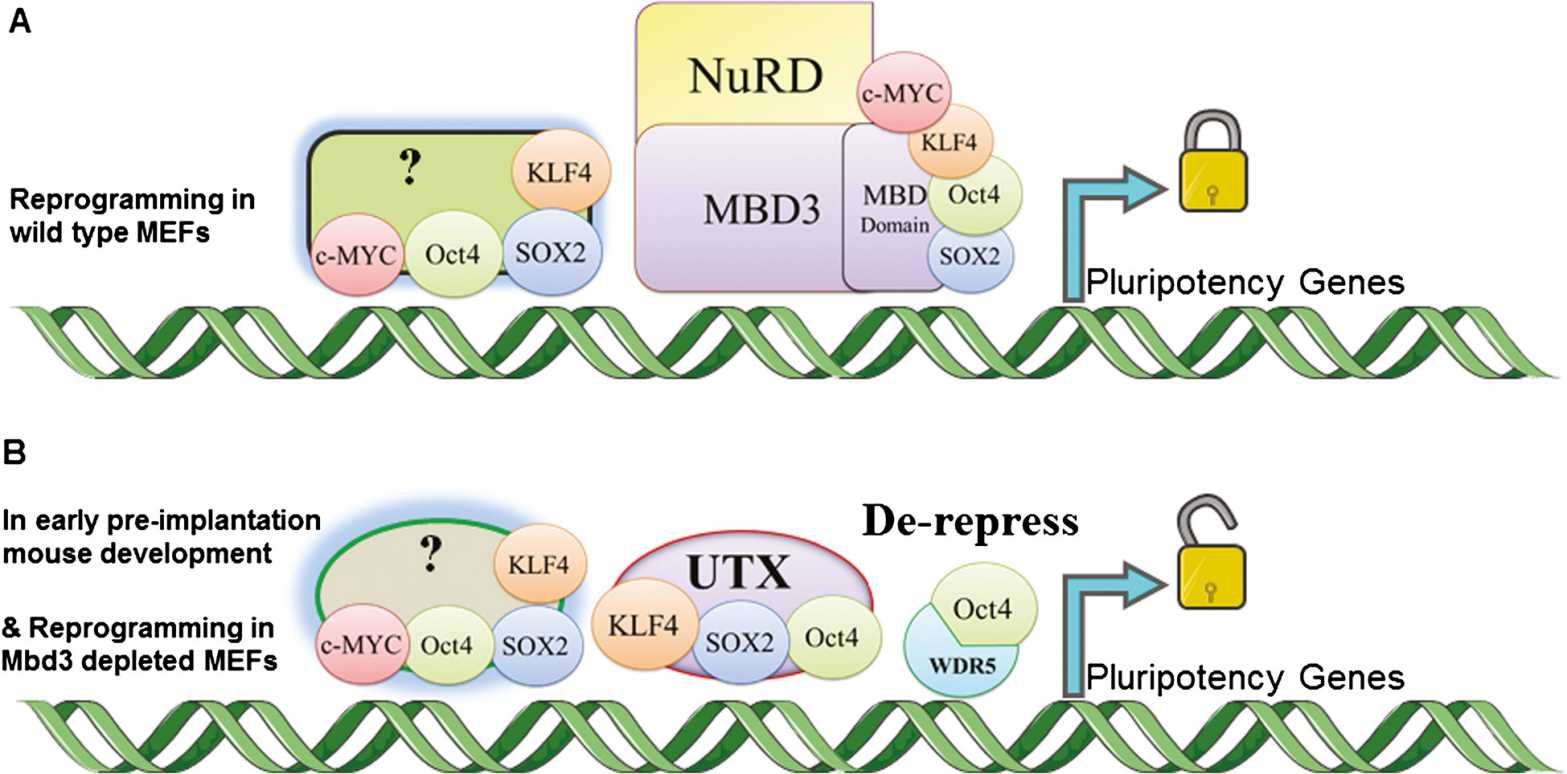
The proposed mechanism by which the Mbd3/NuRD complex acts as a reprogramming barrier. **a** During reprogramming, OSKM proteins bind to the MBD domain of Mbd3 and other repressive complexes (*?*). These interactions lead to inhibition of the expression of pluripotency-related genes and reduction of reprogramming efficiency. **b** Upon *Mbd3* depletion, reprogramming factors are recruited into downstream targets (e.g., pluripotency-related genes) to enhance gene expression. This leads to highly efficient reprogramming to pluripotency. Additionally, WDR5 and UTX are essential in reprogramming of *Mbd3*-depleted MEFs. It has been indicated that *Mbd3* is downregulated during early pre-implantation mouse development and upregulated in the inner cell mass (ICM) and restricts aberrant specification into the trophoblast lineage [9]

Hanna and colleagues reported that before OSKM overexpression, Mbd3 and Chd4 (NuRD components) are not localized to pluripotency factor target genes; however, after OSKM induction, Klf4, Oct4, Sox2, and Esrrb target genes become enriched for Mbd3 and Chd4 binding. Interestingly, these target genes become significantly upregulated upon depletion of *Mbd3*, which indicates that the Mbd3/NuRD complex is a repressor of reprogramming [9]. After OSKM overexpression, Mbd3 binding shows an eightfold increase. This resembles a brake in somatic cells that resists against reprogramming to pluripotency (Fig. 1a). It has been indicated that depletion of *Mbd3* increases Oct4 binding, H3K4me3, and H3K27ac (derepression marks) and decreases H3K27me3 (repressive chromatin mark) during reprogramming [8, 9]. In addition to OSKM expression, Utx and Wdr5 are also essential for reprogramming in *Mbd3*-depleted cells (Fig. 1b) [9].

During reprogramming, exogenous OSKM proteins bind to the MBD domain of Mbd3 and a direct interaction of Mbd3/NuRD and Chd4/NuRD with overexpressed OSKM recruits these complexes into their own promoters and downstream targets of Klf4, Oct4, Sox2, and Esrrb that consequently causes repression of pluripotency genes. NuRD and OSKM cannot assemble a repression complex in the absence of Mbd3, and subsequently, OSKM and downstream targets are activated under the continued expression of exogenous OSKM (Fig. 1b). Accordingly, the Hanna group described this process as a “gas and brakes” paradigm [9].

Surprisingly, dos Santos et al. indicated that overexpression of Mbd3 is not only a barrier but also in conjunction with overexpressed Nanog can increase reprogramming kinetics and efficiency in MEF-derived pre-iPSCs [13]. They demonstrated that overexpressed Nanog interacts with NuRD, and in the presence of overexpressed Mbd3, the Mbd3/NuRD complex enhances reprogramming efficiency of MEF-derived pre-iPSCs and EpiSCs [13]. Contradictory to this finding, Mbd3 has been reported as a barrier of reprogramming in the late stages by silencing *Nanog* [8].

## The CCAAT/enhancer-binding protein-α (C/EBPα)

In attempting to increase the efficiency of reprogramming, the Graf’s laboratory recently reported an efficient method for the reprogramming of mouse-committed B-cell precursors (B cells) into iPSCs [12]. Interestingly, they found that transient expression of CCAAT/enhancer-binding protein-α (C/EBPα) for 18 h followed by OSKM expression induces pluripotent reprogramming in B cells with 95 % efficiency and accelerated kinetics. In detail, direct binding of overexpressed C/EBPα to methylcytosine dioxygenase *Tet2* leads to the upregulation of this gene and then binding of TET2 to the regulatory regions of pluripotency genes. This action leads to oxidation of 5-methylcytosine (5mC) residues into 5-hydroxymethylcytosine (5hmC). This modification induces demethylation, chromatin remodeling, and transcriptional derepression and, subsequently, makes pluripotency gene promoters more accessible for the Oct4 binding. It has been indicated that transient expression of C/EBPα initiates an epithelial-to-mesenchymal transition (EMT), but subsequent OSKM overexpression switched EMT off, and then, MET proceeds efficient iPSC generation. The C/EBPα technique can enhance reprogramming of B cells with 100 % efficiency; however, this approach has been indicated as cell-type specific and inoperative in pluripotent reprogramming of MEFs [12].

## The privileged cell state

Distinct cell types have different requirements to undergo reprogramming into iPSCs, based on the tissues and species from which they are derived as well as in vitro environmental conditions [107]. Surprisingly, a subset of cells has recently been identified in granulocyte monocyte progenitors (GMPs), which is highly competent for non-stochastic reprogramming into iPSCs. Guo et al. named these cells *privileged* cells for pluripotent reprogramming. They demonstrated that privileged cells have an ultrafast cell cycle (~8 h) and can be synchronously reprogrammed with a short latency [7].

It has been demonstrated that there is a direct relation between cell cycle rate and reprogramming efficiency [50]. Therefore, speeding the cell cycle up could induce the emergence of privileged cells [7]. Conversely, Xu et al. indicated that hyperproliferation might have a negative effect on reprogramming efficiency [108].

Interestingly, Guo et al. produced a small population (1–8 %) of ultrafast cycling MEFs from normal MEFs by transient overexpression of Yamanaka factors (OSKM) in a limited time window (6 days) [7], before the establishment of pluripotency. However, there is challenging evidence that indicates transient acquisition of pluripotency during the short-term OSKM treatment [109, 110] Guo et al. showed that pluripotent reprogramming of the ultrafast MEFs is more efficient (~99.7 %) than that of the normal MEFs [7]. These cells are named “lucky” cells, because of their commitment toward an iPSC fate [111]. It is suggested that these lucky cells only adopted a fast and accelerated cell cycle, which is a feature of pluripotent cells, and non-stochasticity is unlikely to occur. Accordingly, the term “privileged somatic cells” for partially pluripotent-committed MEFs seems controversial [111].

Interestingly, it is revealed that depletion of p53 and p57 by a cell cycle acceleration mechanism controls the emergence of ultrafast cycling cells and increases reprogramming efficiency of hematopoietic stem and progenitor cells (HSPCs) as well as Lin^−^/c-Kit^+^/Sca^+^ (LKS) cells [7]. In line with the induction of a “privileged state,” different factors might facilitate the emergence of this state, such as the factors and methods that are discussed in this study.

Collectively, this finding suggests that by identification, stimulation and isolation of ultrafast cycling cells from a known cell line (e.g., stem/progenitor cells) iPSC generation could be achieved in a deterministic level. If this inherent capacity could be stimulated in vivo, it could provide a novel strategy for regenerative biomedicine.

## Combinatorial modulation of barriers and enhancers

Notably, this review considered the removal of barriers as an enhancing strategy. Motivated by the aforementioned discussions, it could be concluded that the simultaneous removal of barriers and activation or administration of enhancers would have a cumulative and maximal effect on improving reprogramming efficiency and kinetics. However, this concept can be effective in the presence of synergism and in the absence of unexpected and neutralizing interactions. For example, knockdown of p53 can override the enhancing effects of P38, IP3K, and AurkA kinase inhibitors [50]. Therefore, some incompatibilities may exist between methods to enhance reprogramming [50].

On the other hand, it has been indicated that particular pathways that act as barriers to reprogramming have interactions and subsequently combinatorial effects to oppose the reprogramming process [80]. For instance, the clathrin-mediated endocytosis and TGF-β signaling pathways have a positive linear interaction during reprogramming that antagonizes reprogramming and subsequently decreases the efficiency [80]. Therefore, inhibition of multiple barriers could have an increasing effect on improving the efficiency. For example, small molecule Pitstop 2 (endocytosis inhibitor) as well as shRNAs for ADAM metallopeptidase domain 29 (ADAM29) and ATF7IP (a chromatin regulator) enhances reprogramming efficiency up to 15-fold in a synergistic manner [80]. Interestingly, as a proof of concept for the increased effects of combinatorial modifications, Vidal and colleagues recently revealed that modulation of specific signaling pathways (Wnt/β-catenin, TGF-β, and MAP kinase) and chromatin state (by ascorbic acid and Kdm2b) synergistically enhances the efficiency of reprogramming to a deterministic level, in a non-stochastic manner and with an accelerated kinetics [20]. This combinatorial method has reached one of the highest efficiencies that are reported for highly efficient (80–100 %) pluripotent reprogramming [20]. Therefore, this finding is the best proof of principle that a combinatorial method of deletion of barriers and activation of enhancers can progress pluripotent reprogramming with high efficiencies and with accelerated kinetics (Table 1).

## Conclusions

The low efficiency and slow kinetics of somatic cell reprogramming are limitations preventing the use of iPSCs in clinic and regenerative medicine [112–114]. Several methods have been used for enhancing reprogramming efficiency. The best strategy is to avoid any genetic manipulation and overexpression of enhancing genes and instead to use small molecule substitutes.

Among the reviewed roadblocks and enhancers, some of them are particularly interesting and significant [7, 9, 12, 13, 115]. Arguably, the most controversial barrier/enhancer of reprogramming is Mbd3 [8, 9, 13], which is extensively discussed in previous sections. Another interesting and potent enhancer is C/EBPα. C/EBPα overexpression is applicable only in B cells and cannot enhance the reprogramming efficiency of other cell types (e.g., fibroblasts) [12]. Therefore, it will be interesting to determine whether conditions exist that permit the application of this factor (or similar factors) in pluripotent reprogramming of other somatic cells. Notably, different somatic cell types have distinct requirements for efficient reprogramming, and a universal strategy for safe and efficient reprogramming in all cell types has yet to be reported.

An interesting report on efficient reprogramming was recently published by Guo and colleagues [7], although there is skepticism about some definitions [111]. Excitingly, they identified a special cell state known as the “privileged” cell state that is more amenable to non-stochastic and highly efficient reprogramming. The privileged state is a situation that both exists naturally and can be attained by alternative means as a dynamic cell state [7]. Surprisingly, “acquired privilege” could be induced by transient overexpression of Yamanaka factors or specific cytokines in MEFs and LKS cells, respectively. However, distinct cell types might need different induction methods [7, 20]. Nevertheless, reprogramming efficiency and its latency varied based on the cell line and the somatic or acquired types of the privileged state [7].

One of the highest efficiencies (~100 %) for pluripotent reprogramming has recently been reported by Vidal and colleagues. They showed that combinatorial modulation of barrier/enhancer signaling pathways and chromatin modifiers strongly facilitates reprogramming in a synchronous and homogenous manner [20]. Remarkably, their finding is the best proof to date that a controlled combinatorial modulation of barriers and enhancers can advance pluripotent reprogramming with 100 % efficiency. A collection of barriers and enhancers are displayed in Table 1. Notably, non-coding RNAs play key roles during somatic cell reprogramming [116, 117] (well discussed by others [118–120]), although this study does not deal with them.

In addition to the intrinsic barriers of reprogramming, environmental conditions play a significant role during reprogramming. For instance, fetal bovine serum (FBS) arrests reprogramming at an intermediate stage by maintaining the somatic cell program and inhibiting the activation of pluripotency genes [81]. In this regard, fine-tuning of the components of the reprogramming medium provides a powerful tool for adjusting the reprogramming rate and efficiency. To this end, small molecules are appropriate alternatives for defining and preparing the best optimized reprogramming strategy [121–123]. In addition to the abovementioned barriers, native transcriptional or gene regulatory networks (TRNs/GRNs) appears to be potential barriers of pluripotent reprogramming and direct reprogramming to different lineages [115, 124-125]. Accordingly, transient disruption of native TRN and consequently, unlocking the cells from the somatic program may provide a novel strategy for enhancing pluripotent reprogramming and direct lineage conversion.

Collectively, the mechanistic insights discussed here on enhancing reprogramming efficiency represent significant progress toward the ultimate goal of a universal, rapid, and highly efficient reprogramming strategy.

## References

[1] Takahashi K, Yamanaka S (2006). Induction of pluripotent stem cells from mouse embryonic and adult fibroblast cultures by defined factors. Cell.

[2] Yu J, Vodyanik MA, Smuga-Otto K, Antosiewicz-Bourget J, Frane JL, Tian S (2007). Induced pluripotent stem cell lines derived from human somatic cells. Science..

[3] Takahashi K, Tanabe K, Ohnuki M, Narita M, Ichisaka T, Tomoda K (2007). Induction of pluripotent stem cells from adult human fibroblasts by defined factors. Cell..

[4] Lu X, Zhao T (2013). Clinical therapy using iPSCs: hopes and challenges. Genomics Proteomics Bioinformatics.

[5] Wernig M, Meissner A, Foreman R, Brambrink T, Ku M, Hochedlinger K (2007). In vitro reprogramming of fibroblasts into a pluripotent ES-cell-like state. Nature..

[6] Jiang Z, Han Y, Cao X (2014). Induced pluripotent stem cell (iPSCs) and their application in immunotherapy. Cell Mol Immunol.

[7] Guo S, Zi X, Schulz VP, Cheng J, Zhong M, Koochaki SH (2014). Nonstochastic reprogramming from a privileged somatic cell state. Cell..

[8] Luo M, Ling T, Xie W, Sun H, Zhou Y, Zhu Q (2013). NuRD blocks reprogramming of mouse somatic cells into pluripotent stem cells. Stem Cells..

[9] Rais Y, Zviran A, Geula S, Gafni O, Chomsky E, Viukov S (2013). Deterministic direct reprogramming of somatic cells to pluripotency. Nature..

[10] Worringer KA, Rand TA, Hayashi Y, Sami S, Takahashi K, Tanabe K (2014). The let-7/LIN-41 pathway regulates reprogramming to human induced pluripotent stem cells by controlling expression of prodifferentiation genes. Cell Stem Cell..

[11] Banito A, Rashid ST, Acosta JC, Li S, Pereira CF, Geti I (2009). Senescence impairs successful reprogramming to pluripotent stem cells. Genes Dev..

[12] Di Stefano B, Sardina JL, van Oevelen C, Collombet S, Kallin EM, Vicent GP (2014). C/EBPalpha poises B cells for rapid reprogramming into induced pluripotent stem cells. Nature..

[13] dos Santos RL, Tosti L, Radzisheuskaya A, Caballero Isabel M, Kaji K, Hendrich B (2014). MBD3/NuRD facilitates induction of pluripotency in a context-dependent manner. Cell Stem Cell..

[14] Hasegawa Y, Tang D, Takahashi N, Hayashizaki Y, Forrest AR, The Fantom C (2014). CCL2 enhances pluripotency of human induced pluripotent stem cells by activating hypoxia related genes. Sci Report..

[15] Wang L, Du Y, Ward JM, Shimbo T, Lackford B, Zheng X (2014). INO80 facilitates pluripotency gene activation in embryonic stem cell self-renewal, reprogramming, and blastocyst development. Cell Stem Cell..

[16] Mathew R, Jia W, Sharma A, Zhao Y, Clarke LE, Cheng X (2010). Robust activation of the human but not mouse telomerase gene during the induction of pluripotency. Faseb J..

[17] Yu J, Hu K, Smuga-Otto K, Tian S, Stewart R (2009). Slukvin, et al. Human induced pluripotent stem cells free of vector and transgene sequences. Science.

[18] Wang W, Yang J, Liu H, Lu D, Chen X, Zenonos Z (2011). Rapid and efficient reprogramming of somatic cells to induced pluripotent stem cells by retinoic acid receptor gamma and liver receptor homolog 1. Proc Natl Acad Sci..

[19] Polo JM, Liu S, Figueroa ME, Kulalert W, Eminli S, Tan KY (2010). Cell type of origin influences the molecular and functional properties of mouse induced pluripotent stem cells. Nat Biotechnol..

[20] Vidal SE, Amlani B, Chen T, Tsirigos A, Stadtfeld M (2014). Combinatorial modulation of signaling pathways reveals cell-type-specific requirements for highly efficient and synchronous iPSC reprogramming. Stem Cell Reports..

[21] Montserrat N, Nivet E, Sancho-Martinez I, Hishida T, Kumar S, Miquel L (2013). Reprogramming of human fibroblasts to pluripotency with lineage specifiers. Cell Stem Cell..

[22] Shu J, Wu C, Wu Y, Li Z, Shao S, Zhao W (2013). Induction of pluripotency in mouse somatic cells with lineage specifiers. Cell..

[23] Popowski M, Templeton TD, Lee B-K, Rhee C, Li H, Miner C (2014). Bright/Arid3A acts as a barrier to somatic cell reprogramming through direct regulation of Oct4, Sox2, and Nanog. Stem Cell Reports..

[24] Maekawa M, Yamaguchi K, Nakamura T, Shibukawa R, Kodanaka I, Ichisaka T (2011). Direct reprogramming of somatic cells is promoted by maternal transcription factor GLIS1. Nature..

[25] Takahashi K, Tanabe K, Ohnuki M, Narita M, Sasaki A, Yamamoto M (2014). Induction of pluripotency in human somatic cells via a transient state resembling primitive streak-like mesendoderm. Nat Commun..

[26] Herrscher RF, Kaplan MH, Lelsz DL, Das C, Scheuermann R, Tucker PW (1995). The immunoglobulin heavy-chain matrix-associating regions are bound by Bright: a B cell-specific trans-activator that describes a new DNA-binding protein family. Genes Dev..

[27] Wilsker D, Patsialou A, Dallas PB, Moran E (2002). ARID proteins: a diverse family of DNA binding proteins implicated in the control of cell growth, differentiation, and development. Cell Growth Differ..

[28] An G, Miner CA, Nixon JC, Kincade PW, Bryant J, Tucker PW (2010). Loss of Bright/ARID3a function promotes developmental plasticity. Stem Cells..

[29] Rasmussen Mikkel A, Holst B, Tümer Z, Johnsen Mads G, Zhou S, Stummann Tina C (2014). Transient p53 suppression increases reprogramming of human fibroblasts without affecting apoptosis and DNA damage. Stem Cell Reports..

[30] Huangfu D, Osafune K, Maehr R, Guo W, Eijkelenboom A, Chen S (2008). Induction of pluripotent stem cells from primary human fibroblasts with only Oct4 and Sox2. Nat Biotechnol..

[31] Aksoy I, Stanton LW (2013). Pluripotency-regulating networks provide basis for reprogramming. Curr Mol Med.

[32] Wernig M, Meissner A, Cassady JP, Jaenisch R (2008). c-Myc is dispensable for direct reprogramming of mouse fibroblasts. Cell Stem Cell.

[33] Aksoy I, Jauch R, Eras V (2013). Chng W-bA, Chen J, Divakar U, et al. Sox transcription factors require selective interactions with Oct4 and specific transactivation functions to mediate reprogramming. Stem Cells.

[34] Ng CK, Li NX, Chee S, Prabhakar S, Kolatkar PR, Jauch R (2012). Deciphering the Sox-Oct partner code by quantitative cooperativity measurements. Nucleic Acids Res..

[35] Wang Y, Chen J, Hu JL, Wei XX, Qin D, Gao J (2011). Reprogramming of mouse and human somatic cells by high-performance engineered factors. EMBO Rep..

[36] Jauch R, Aksoy I, Hutchins AP, Ng CK, Tian XF, Chen J (2011). Conversion of Sox17 into a pluripotency reprogramming factor by reengineering its association with Oct4 on DNA. Stem Cells..

[37] Lamouille S, Xu J, Derynck R (2014). Molecular mechanisms of epithelial-mesenchymal transition. Nat Rev Mol Cell Biol.

[38] Li R, Liang J, Ni S, Zhou T, Qing X, Li H (2010). A mesenchymal-to-epithelial transition initiates and is required for the nuclear reprogramming of mouse fibroblasts. Cell Stem Cell..

[39] Sakurai K, Talukdar I, Patil Veena S, Dang J, Li Z, Chang K-Y (2014). Kinome-wide functional analysis highlights the role of cytoskeletal remodeling in somatic cell reprogramming. Cell Stem Cell..

[40] Samavarchi-Tehrani P, Golipour A, David L (2010). Sung H-k, Beyer TA, Datti A, et al. Functional genomics reveals a BMP-driven mesenchymal-to-epithelial transition in the initiation of somatic cell reprogramming. Cell Stem Cell.

[41] Maherali N, Hochedlinger K (2009). Tgfβ signal inhibition cooperates in the induction of iPSCs and replaces Sox2 and cMyc. Curr Biol.

[42] Ichida JK, Blanchard J, Lam K, Son EY, Chung JE, Egli D (2009). A small-molecule inhibitor of tgf-Beta signaling replaces sox2 in reprogramming by inducing nanog. Cell Stem Cell..

[43] Lin T, Ambasudhan R, Yuan X, Li W, Hilcove S, Abujarour R (2009). A chemical platform for improved induction of human iPSCs. Nat Methods..

[44] Brosh R, Assia-Alroy Y, Molchadsky A, Bornstein C, Dekel E, Madar S (2013). p53 counteracts reprogramming by inhibiting mesenchymal-to-epithelial transition. Cell Death Differ..

[45] Unternaehrer Juli J, Zhao R, Kim K, Cesana M, Powers John T, Ratanasirintrawoot S (2014). The epithelial-mesenchymal transition factor SNAIL paradoxically enhances reprogramming. Stem Cell Reports..

[46] Ying QL, Wray J, Nichols J, Batlle-Morera L, Doble B, Woodgett J (2008). The ground state of embryonic stem cell self-renewal. Nature..

[47] Silva J, Barrandon O, Nichols J, Kawaguchi J, Theunissen TW, Smith A (2008). Promotion of reprogramming to ground state pluripotency by signal inhibition. PLoS Biol..

[48] Theunissen TW, van Oosten AL, Castelo-Branco G, Hall J, Smith A, Silva JC (2011). Nanog overcomes reprogramming barriers and induces pluripotency in minimal conditions. Curr Biol..

[49] Takashima Y, Guo G, Loos R, Nichols J, Ficz G, Krueger F (2014). Resetting transcription factor control circuitry toward ground-state pluripotency in human. Cell..

[50] Li Z, Rana TM (2012). A kinase inhibitor screen identifies small-molecule enhancers of reprogramming and iPS cell generation. Nat Commun.

[51] Vousden KH (2000). p53: death star. Cell.

[52] Kawamura T, Suzuki J, Wang YV, Menendez S, Morera LB, Raya A (2009). Linking the p53 tumour suppressor pathway to somatic cell reprogramming. Nature..

[53] Zhao Y, Yin X, Qin H, Zhu F, Liu H, Yang W (2008). Two supporting factors greatly improve the efficiency of human iPSC generation. Cell Stem Cell..

[54] Yulin X, Lizhen L, Lifei Z, Shan F, Ru L, Kaimin H (2012). Efficient generation of induced pluripotent stem cells from human bone marrow mesenchymal stem cells. Folia Biol..

[55] Marion RM, Strati K, Li H, Murga M, Blanco R, Ortega S (2009). A p53-mediated DNA damage response limits reprogramming to ensure iPS cell genomic integrity. Nature..

[56] Hong H, Takahashi K, Ichisaka T, Aoi T, Kanagawa O, Nakagawa M (2009). Suppression of induced pluripotent stem cell generation by the p53–p21 pathway. Nature..

[57] Li H, Collado M, Villasante A, Strati K, Ortega S, Canamero M (2009). The Ink4/Arf locus is a barrier for iPS cell reprogramming. Nature..

[58] Utikal J, Polo JM, Stadtfeld M, Maherali N, Kulalert W, Walsh RM (2009). Immortalization eliminates a roadblock during cellular reprogramming into iPS cells. Nature..

[59] Hanna J, Saha K, Pando B, van Zon J, Lengner CJ, Creyghton MP (2009). Direct cell reprogramming is a stochastic process amenable to acceleration. Nature..

[60] Yang CS, Chang KY, Rana TM (2014). Genome-wide functional analysis reveals factors needed at the transition steps of induced reprogramming. Cell Rep.

[61] Zhao T, Xu Y (2010). p53 and stem cells: new developments and new concerns. Trends Cell Biol.

[62] Okita K, Matsumura Y, Sato Y, Okada A, Morizane A, Okamoto S (2011). A more efficient method to generate integration-free human iPS cells. Nat Methods..

[63] Okita K, Yamakawa T, Matsumura Y, Sato Y, Amano N, Watanabe A (2013). An efficient nonviral method to generate integration-free human-induced pluripotent stem cells from cord blood and peripheral blood cells. Stem Cells..

[64] Chen Z, Zhao T, Xu Y (2012). The genomic stability of induced pluripotent stem cells. Protein & cell.

[65] Lake BB, Fink J, Klemetsaune L, Fu X, Jeffers JR, Zambetti GP (2012). Context-dependent enhancement of induced pluripotent stem cell reprogramming by silencing Puma. Stem Cells..

[66] Menendez S, Camus S, Izpisua Belmonte JC (2010). p53: guardian of reprogramming. Cell Cycle.

[67] Komarov PG, Komarova EA, Kondratov RV, Christov-Tselkov K, Coon JS, Chernov MV (1999). A chemical inhibitor of p53 that protects mice from the side effects of cancer therapy. Science..

[68] Lluis F, Pedone E, Pepe S, Cosma MP (2008). Periodic activation of Wnt/beta-catenin signaling enhances somatic cell reprogramming mediated by cell fusion. Cell Stem Cell..

[69] Ho R, Papp B, Hoffman JA, Merrill BJ, Plath K (2013). Stage-specific regulation of reprogramming to induced pluripotent stem cells by Wnt signaling and T cell factor proteins. Cell Rep..

[70] Aulicino F, Theka I, Ombrato L, Lluis F, Cosma MP (2014). Temporal perturbation of the Wnt signaling pathway in the control of cell reprogramming is modulated by TCF1. Stem Cell Reports..

[71] Zhang P, Chang W-H, Fong B, Gao F, Liu C, Al Alam D (2014). Regulation of iPS cell induction by Wnt/β-catenin signaling. J Biol Chem..

[72] Lian X, Hsiao C, Wilson G, Zhu K, Hazeltine LB, Azarin SM, et al. Robust cardiomyocyte differentiation from human pluripotent stem cells via temporal modulation of canonical Wnt signaling. Proc Natl Acad Sci USA. 2012;109:E1848–57. 10.1073/pnas.1200250109 PMC3390875 22645348

[73] Lian X, Zhang J, Azarin SM, Zhu K, Hazeltine LB, Bao X (2013). Directed cardiomyocyte differentiation from human pluripotent stem cells by modulating Wnt/beta-catenin signaling under fully defined conditions. Nat Protoc..

[74] Murayama H, Masaki H, Sato H, Hayama T, Yamaguchi T, Nakauchi H (2014). Successful reprogramming of epiblast stem cells by blocking nuclear localization of β-catenin. Stem Cell Reports..

[75] Johnson R, Halder G (2014). The two faces of Hippo: targeting the Hippo pathway for regenerative medicine and cancer treatment. Nat Rev Drug Discov.

[76] Lian I, Kim J, Okazawa H, Zhao J, Zhao B, Yu J (2010). The role of YAP transcription coactivator in regulating stem cell self-renewal and differentiation. Genes Dev..

[77] Qin H, Blaschke K, Wei G, Ohi Y, Blouin L, Qi Z (2012). Transcriptional analysis of pluripotency reveals the Hippo pathway as a barrier to reprogramming. Hum Mol Genet..

[78] Buckley SM, Aranda-Orgilles B, Strikoudis A, Apostolou E, Loizou E, Moran-Crusio K (2012). Regulation of pluripotency and cellular reprogramming by the ubiquitin-proteasome system. Cell Stem Cell..

[79] Vilchez D, Boyer L, Morantte I, Lutz M, Merkwirth C, Joyce D (2012). Increased proteasome activity in human embryonic stem cells is regulated by PSMD11. Nature..

[80] Qin H, Diaz A, Blouin L, Lebbink RJ, Patena W, Tanbun P (2014). Systematic identification of barriers to human iPSC generation. Cell..

[81] Chen JK, Liu H, Liu J, Qi J, Wei B, Yang JQ (2013). H3K9 methylation is a barrier during somatic cell reprogramming into iPSCs. Nat Genet..

[82] Onder TT, Kara N, Cherry A, Sinha AU, Zhu N, Bernt KM (2012). Chromatin-modifying enzymes as modulators of reprogramming. Nature..

[83] Hanna J, Markoulaki S, Schorderet P, Carey BW, Beard C, Wernig M (2008). Direct reprogramming of terminally differentiated mature B lymphocytes to pluripotency. Cell..

[84] Mikkelsen TS, Hanna J, Zhang X, Ku M, Wernig M, Schorderet P (2008). Dissecting direct reprogramming through integrative genomic analysis. Nature..

[85] Pasque V, Radzisheuskaya A, Gillich A, Halley-Stott RP, Panamarova M, Zernicka-Goetz M (2012). Histone variant macroH2A marks embryonic differentiation in vivo and acts as an epigenetic barrier to induced pluripotency. J Cell Sci..

[86] Gaspar-Maia A, Qadeer ZA, Hasson D, Ratnakumar K, Leu NA, Leroy G (2013). MacroH2A histone variants act as a barrier upon reprogramming towards pluripotency. Nat Commun..

[87] Wang T, Chen K, Zeng X, Yang J, Wu Y, Shi X (2011). The histone demethylases Jhdm1a/1b enhance somatic cell reprogramming in a vitamin-C-dependent manner. Cell Stem Cell..

[88] He J, Kallin EM, Tsukada Y, Zhang Y (2008). The H3K36 demethylase Jhdm1b/Kdm2b regulates cell proliferation and senescence through p15(Ink4b). Nat Struct Mol Biol..

[89] Tzatsos A, Pfau R, Kampranis SC, Tsichlis PN. Ndy1/KDM2B immortalizes mouse embryonic fibroblasts by repressing the Ink4a/Arf locus. Proc Natl Acad Sci USA. 2009;106:2641–6. 10.1073/pnas.0813139106 PMC2650317 19202064

[90] Liao B, Bao X, Liu L, Feng S, Zovoilis A, Liu W (2011). MicroRNA cluster 302–367 enhances somatic cell reprogramming by accelerating a mesenchymal-to-epithelial transition. J Biol Chem..

[91] Subramanyam D, Lamouille S, Judson RL, Liu JY, Bucay N, Derynck R (2011). Multiple targets of miR-302 and miR-372 promote reprogramming of human fibroblasts to induced pluripotent stem cells. Nat Biotechnol..

[92] Liang G, Taranova O, Xia K, Zhang Y (2010). Butyrate promotes induced pluripotent stem cell generation. J Biol Chem..

[93] Mali P, Chou BK, Yen J, Ye Z, Zou J, Dowey S (2010). Butyrate greatly enhances derivation of human induced pluripotent stem cells by promoting epigenetic remodeling and the expression of pluripotency-associated genes. Stem Cells..

[94] Huangfu D, Maehr R, Guo W, Eijkelenboom A, Snitow M, Chen AE (2008). Induction of pluripotent stem cells by defined factors is greatly improved by small-molecule compounds. Nat Biotech..

[95] Zhang Z, Wu WS (2013). Sodium butyrate promotes generation of human induced pluripotent stem cells through induction of the miR302/367 cluster. Stem Cells Dev.

[96] Li X, Li L, Pandey R, Byun JS, Gardner K, Qin Z (2012). The histone acetyltransferase MOF is a key regulator of the embryonic stem cell core transcriptional network. Cell Stem Cell..

[97] Mu X, Yan S, Fu C, Wei A (2015). The histone acetyltransferase MOF promotes induces generation of pluripotent stem cells. Cell Reprogram..

[98] Buganim Y, Faddah DA, Jaenisch R (2013). Mechanisms and models of somatic cell reprogramming. Nat Rev Genet.

[99] Theunissen Thorold W, Jaenisch R (2014). Molecular control of induced pluripotency. Cell Stem Cell.

[100] Laugesen A, Helin K (2014). Chromatin repressive complexes in stem cells, development, and cancer. Cell Stem Cell.

[101] Vierbuchen T, Ostermeier A, Pang ZP, Kokubu Y, Sudhof TC, Wernig M. Direct conversion of fibroblasts to functional neurons by defined factors. Nature. 2010;463:1035–41. 10.1038/nature08797 PMC2829121 20107439

[102] Ieda M, Fu JD, Delgado-Olguin P, Vedantham V, Hayashi Y, Bruneau BG (2010). Direct reprogramming of fibroblasts into functional cardiomyocytes by defined factors. Cell..

[103] Fidalgo M, Faiola F, Pereira C-F, Ding J, Saunders A, Gingold J (2012). Zfp281 mediates Nanog autorepression through recruitment of the NuRD complex and inhibits somatic cell reprogramming. Proc Natl Acad Sci..

[104] Bertone P, Hendrich B, Silva JCR. Mbd3 and deterministic reprogramming. bioRxiv*.* 2015. doi:10.1101/013904.

[105] Zviran A, Rais Y, Mor N, Novershtern N, Hanna JH. Mbd3/NuRD is a key inhibitory module during the induction and maintenance of naïve pluripotency. biorxiv*.* 2015. doi:10.1101/013961.

[106] Lujan E, Zunder ER, Ng YH, Goronzy IN, Nolan GP, Wernig M (2015). Early reprogramming regulators identified by prospective isolation and mass cytometry. Nature..

[107] Liebau S, Mahaddalkar PU, Kestler HA, Illing A, Seufferlein T, Kleger A (2013). A hierarchy in reprogramming capacity in different tissue microenvironments: what we know and what we need to know. Stem Cells Dev..

[108] Xu Y, Wei X, Wang M, Zhang R, Fu Y, Xing M (2013). Proliferation rate of somatic cells affects reprogramming efficiency. J Biol Chem..

[109] Maza I, Caspi I, Zviran A, Chomsky E, Rais Y, Viukov S (2015). Transient acquisition of pluripotency during somatic cell transdifferentiation with iPSC reprogramming factors. Nat Biotech..

[110] Bar-Nur O, Verheul C, Sommer AG, Brumbaugh J, Schwarz BA, Lipchina I (2015). Lineage conversion induced by pluripotency factors involves transient passage through an iPSC stage. Nat Biotech..

[111] Zviran A, Hanna J (2014). Lucky iPSCs. Genome Biol.

[112] Yamanaka S (2007). Strategies and new developments in the generation of patient-specific pluripotent stem cells. Cell Stem Cell.

[113] Chen J, Liu J, Chen Y, Yang J, Chen J, Liu H (2011). Rational optimization of reprogramming culture conditions for the generation of induced pluripotent stem cells with ultra-high efficiency and fast kinetics. Cell Res..

[114] Masip M, Veiga A, Izpisua Belmonte JC, Simon C (2010). Reprogramming with defined factors: from induced pluripotency to induced transdifferentiation. Mol Hum Reprod..

[115] Tomaru Y, Hasegawa R, Suzuki T, Sato T, Kubosaki A, Suzuki M (2014). A transient disruption of fibroblastic transcriptional regulatory network facilitates trans-differentiation. Nucleic Acids Res.

[116] Loewer S, Cabili MN, Guttman M, Loh Y-H, Thomas K, Park IH (2010). Large intergenic non-coding RNA-RoR modulates reprogramming of human induced pluripotent stem cells. Nat Genet..

[117] Neveu P, Kye MJ, Qi S, Buchholz DE, Clegg DO, Sahin M (2010). MicroRNA profiling reveals two distinct p53-related human pluripotent stem cell states. Cell Stem Cell..

[118] González F, Boué S, Belmonte JCI (2011). Methods for making induced pluripotent stem cells: reprogramming à la carte. Nat Rev Genet.

[119] Huo JS, Zambidis ET (2013). Pivots of pluripotency: the roles of non-coding RNA in regulating embryonic and induced pluripotent stem cells. Biochim Biophys Acta Gen Subj.

[120] Jia W, Chen W, Kang J (2013). The functions of microRNAs and long non-coding RNAs in embryonic and induced pluripotent stem cells. Genomics Proteomics Bioinformatics.

[121] Li W, Jiang K, Ding S (2012). Concise review: a chemical approach to control cell fate and function. Stem Cells.

[122] Li W, Jiang K, Wei W, Shi Y, Ding S (2013). Chemical approaches to studying stem cell biology. Cell Res..

[123] Li W, Li K, Wei W, Ding S (2013). Chemical approaches to stem cell biology and therapeutics. Cell Stem Cell..

[124] Cahan P, Li H, Morris Samantha A (2014). Lummertz da Rocha E, Daley George Q, Collins James J. Cell net: network biology applied to stem cell engineering. Cell.

[125] Morris Samantha A, Cahan P, Li H, Zhao Anna M, San Roman Adrianna K, Shivdasani Ramesh A, et al. Dissecting engineered cell types and enhancing cell fate conversion via cell net. Cell. 2014;158:889–902. 10.1016/j.cell.2014.07.021 PMC4291075 25126792

